# Correlation between spleen density and prognostic outcomes in patients with colorectal cancer after curative resection

**DOI:** 10.1186/s12885-024-12208-7

**Published:** 2024-04-06

**Authors:** Yunzhou Xiao, Xiaoting Wen, Yingying Ying, Xiaoyan Zhang, Luyao Li, Zhongchu Wang, Miaoguang Su, Shouliang Miao

**Affiliations:** 1 Department of Radiology, PingYang People’s Hospital, Wenzhou Medical University, Wenzhou, 325400 China; 2 Department of Obstetrics, PingYang People’s Hospital, Wenzhou Medical University, Wenzhou, 325400 China; 3 Department of Radiology, The First Affiliated Hospital of Wenzhou Medical University, Wenzhou, 325000 China

**Keywords:** Spleen density, Nomogram, Prognosis, Colorectal cancer, Overall survival, Disease-free survival

## Abstract

**Objective:**

The objective of this study was to investigate the correlation between spleen density and the prognostic outcomes of patients who underwent curative resection for colorectal cancer (CRC).

**Methods:**

The clinical data of patients who were diagnosed with CRC and underwent radical resection were retrospectively analyzed. Spleen density was determined using computed tomography. Analysis of spleen density in relation to overall survival (OS) and disease-free survival (DFS) utilizing the Kaplan–Meier method. Univariate and multivariate Cox regression models were used to screen for independent prognostic factors, and a nomogram was constructed to predict OS and DFS. Moreover, internally validated using a bootstrap resamplling method.

**Results:**

Two hundred twelve patients were included, of whom 23 (10.85%) were defined as having a diffuse reduction of spleen density (DROSD) based on diagnostic cutoff values (spleen density≦37.00HU). Kaplan–Meier analysis indicated that patients with DROSD had worse OS and DFS than those non-DROSD (*P* < 0.05). Multivariate Cox regression analysis revealed that DROSD, carbohydrate antigen 199 (CA199) > 37 U/mL, tumor node metastasis (TNM) stage III-IV, laparoscopy-assisted operation and American Society of Anesthesiology (ASA) score were independent risk factors for 3-year DFS. DROSD, CA199 > 37 U/mL, TNM stage III-IV, hypoalbuminemia, laparoscopy-assisted operation and ASA score were chosen as predictors of for 3-year OS. Nomograms showed satisfactory accuracy in predicting OS and DFS using calibration curves, decision curve analysis and bootstrap resamplling method.

**Conclusion:**

Patients with DROSD who underwent curative resection have worse 3-year DFS and OS. The nomogram demonstrated good performance, particularly in predicting 3-year DFS with a net clinical benefit superior to well-established risk calculator.

**Supplementary Information:**

The online version contains supplementary material available at 10.1186/s12885-024-12208-7.

## Introduction

Colorectal cancer (CRC) has become the third most common malignancy worldwide, and is a leading cause of cancer-related deaths, with the second mortality rate [1, 2]. At present, surgery is the most important and decisive method for the treatment of CRC [3]. Although advances in medical treatment have gradually improved patient survival, colorectal cancer remains a fatal disease with poor prognosis [4, 5]. Consequently, it is imperative to investigate the pertinent factors influencing the prognosis of colorectal cancer and develop a comprehensive model. Several studies have demonstrated that certain systemic inflammatory factors, including platelet-to-lymphocyte ratio [6], C-reactive protein [7], and neutrophil-to-lymphocyte ratio [8], can serve as prognostic indicators for patients with colorectal cancer. However, none of these factors, either individually or in combination, are capable of accurately identifying high-risk patients. Hence, the discovery of new predictive markers may help identify high-risk patients, increase the predictability of prognosis, and improve treatment rates.

Immunologic functions are closely related to the prognosis of colorectal cancer [9], and the spleen is the largest secondary lymphoid organ in the body and hosts a wide range of immunologic functions alongside its roles in hematopoiesis and red blood cell clearance [10]. Diffuse reduction of spleen density (DROSD) is an imaging manifestation in the abdominal computed tomography (CT) that reflects the immune status of the spleen. Previous study has also demonstrated that DROSD is associated with a poor prognosis in acute pancreatitis [11]. In addition, DROSD has been shown to be associated with poor prognosis in gastric cancer [12], intrahepatic cholangiocarcinoma [13] and acute mesenteric ischemia [14]. This phenomenon has been observed in some patients with CRC in our daily clinical practice. Nevertheless, there is still a scarcity of predictive values for DROSD in CRC prognosis.

This investigation aimed to determine the prognostic significance of DROSD in CRC patients who have undergone curative surgery. Additionally, a nomogram model was created to assist in clinical decision-making.

## Materials and methods

### Patient selection

Consecutive patients with colorectal cancer who underwent radical resection from May 2014 to December 2019 were included in this study, with the following criteria: abdominal non-contrast CT scan was performed within 1 month before surgery. The exclusion criteria were as follows: (1) patients with a history of splenic and hematological diseases; (2) patients with a history of previous anticancer treatment or other malignant tumors; (3) incomplete medical records; (4) loss of follow-up after discharge. This study was conducted in accordance with the Declaration of Helsinki and ethical approval was obtained from the Ethics Committee of PingYang People's Hospital. The requirement of patient informed consent was waived owing to the retrospective nature of the study.

### Data collection

Data on preoperative patient characteristics and postoperative outcomes were accessed from electronic medical records. An analysis was conducted retrospectively on the following variables: (1) patient demographics and clinical characteristics, such as age, sex, body mass index (BMI), hemoglobin levels (defined as anemia if < 120 g/L in men and < 110 g/L in women), plasma albumin levels (defined as hypoalbuminemia if < 35 g/L), white blood cell (WBC) count, Charlson Comorbidity Index (CCI) score [15], American Society of Anesthesiology (ASA) scores, Nutritional Risk Screening 2002 (NRS 2002) scores, history of prior abdominal surgery, tumor node metastasis (TNM) stage, chemotherapy, tumor site, histological type, tumor size and radiotherapy; (2) surgical details, including use of epidural anesthesia, laparoscopy-assisted operation, estimated blood loss, surgical duration, combined resection and creation of enterostomy; (3) Short-term postoperative outcomes, such as length of hospital stay and occurrence of complications within 30 days. Complications were categorized according to the Clavien-Dindo classification as Clavien-Dindo 0-II and Clavien-Dindo III-V, with the latter considered significant [16].

### Measurement of the spleen density

**Fig. 1:**
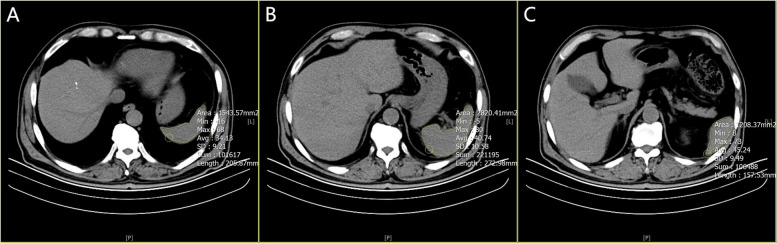
Abdominal CT scan of a colorectal cancer patient with spleen density. The CT value of spleen at the (**A**) upper pole level, (**B**) the hilum level, and the (**C**) lower pole level was 34.13, 40.74, and 45.24 HU, respectively

Spleen density was measured on the images of preoperative abdominal non-contrast CT scans in our hospital, using the same scanning parameters (Tube voltage 120 kV; tube rotation time 750 ms; tube current 50 mA; layer spacing 5 mm; and layer thickness 5 mm). As shown in Fig. 1, the CT values (Hounsfield units, HU) at the levels of upper pole, hilum and lower pole of the spleen were measured using a special processing system (version 3.0.11.3 BN1732 bit; INFINITT Healthcare Co. Ltd., Seoul, South Korea). Spleen density was defined as the mean of three measurements of the CT value of the spleen. To minimize systematic error, two investigators blinded to all clinical and surgical characteristics were trained to measure and evaluate spleen density.

A “survminer” package in R software to determine the best cut-off of spleen density for survival analysis. Based on this cut-off value, patients were then divided into DROSD and non-DROSD groups.

### Follow-up

All patients had to return within the first month after surgery and undergo the necessary examinations. Thereafter, the patients were reviewed every 3 months for the first 2 years, every 6 months for the third year, and then once a year. The Follow-up program consisted of physical examination, laboratory examination, ultrasound and/or CT and/or endoscopy. Overall survival (OS) and disease-free survival (DFS) were the primary endpoints. OS was calculated from the date of surgery to the date of patient death or last follow-up. DFS was defined from the date of surgery to the date of first CRC recurrence, death, or last follow-up visit. Patient follow-up data, obtained mainly from medical records and telephone follow-up. The date of the last follow-up was December 2022.

### Statistical analyses

The Kolmogorov–Smirnov test was used to assess whether continuous data were normally distributed (*P*-value > 0.05). Continuous data that followed a normal distribution were displayed as mean ± standard deviation, and group comparisons were conducted with the Student’s t-test. Variables that did not conform to a normal distribution were represented as median along with the interquartile range (IQR), and the Mann–Whitney U-test was utilized. Comparisons of categorical data were carried out using either the chi-squared test or Fisher’s exact probability test. The Kaplan–Meier method and log-rank test were used to estimate OS and DFS among the different groups. Variables with *P*-value < 0.1 in univariate Cox regression analysis were included in subsequent multivariate forward–backward stepwise Cox regression based on minimum Akaike information criteria (AIC) to evaluate prognostic factors associated with OS and DFS. A nomogram was created based on the results of multivariate analysis and internally validated using bootstrapping method with 1,000 resamples. All tests were 2-sided, and a *P*-value < 0.05 indicated statistical significance. The statistical analyses were performed using the R version 4.3.0 software programs.

## Results

### Patient characteristics

A total of 238 consecutive patients with colorectal cancer who underwent radical resection between May 2014 and December 2019 were shortlisted from the hospital database. Of these, 212 patients who met the inclusion criteria constituted the study cohort. The median follow-up duration was 59.00(95% CI 55.24–62.76) months. The 1-, 2- and 3-year OS rates were 95.70%, 85.30% and 82.50%, respectively. The 1-, 2- and 3-year DFS rates were 86.30%, 78.80% and 74.00%, respectively. Cutoff value for DROSD associated with DFS was 37.00 HU. Using this cutoff value, 23 (10.85%) patients were found to have DROSD. As shown in Table 1, patients with DROSD had a higher CEA, no radiotherapy and chemotherapy, previous abdominal surgery, no laparoscopy-assisted operation and poorer Clavien-Dindo Classification than that those without DROSD (all *P* < 0.05).

**Table 1:** Patient Demographic and Clinical Characteristics

Factors	Total (*n* = 212)	non-DROSD (*n* = 189)	DROSD (*n* = 23)	*P*-values
Age (years), Mean ± SD	69.45 ± 10.63	69.21 ± 10.56	71.39 ± 11.22	0.354
BMI (kg/cm^2^), Mean ± SD	22.19 ± 3.32	22.04 ± 3.12	23.40 ± 4.53	0.063
Surgical duration (min), Mean ± SD	174.21 ± 48.97	175.35 ± 50.05	164.78 ± 38.66	0.329
Length of postoperative hospital stay (day), Mean ± SD	16.49 ± 10.75	16.30 ± 11.23	18.09 ± 5.30	0.452
Gender, n (%)				0.137
Male	140 (66.04)	128 (67.72)	12 (52.17)	
Female	72 (33.96)	61 (32.28)	11 (47.83)	
Tumor size (cm), n (%)				0.324
<4	75 (35.38)	69 (36.51)	6 (26.09)	
≧4	137 (64.62)	120 (63.49)	17 (73.91)	
Tumor location, n (%)				0.545
Rectum	98 (46.23)	86 (45.50)	12 (52.17)	
Colon	114 (53.77)	103 (54.50)	11 (47.83)	
TNM, n (%)				0.727
I	31 (14.62)	29 (15.34)	2 (8.70)	
II	99 (46.7)	89 (47.09)	10 (43.48)	
III	70 (33.02)	60 (31.75)	10 (43.48)	
IV	12 (5.66)	11 (5.82)	1 (4.35)	
Histologic type, n (%)				0.371
Differentiated^a^	208 (98.11)	186 (98.41)	22 (95.65)	
Undifferentiated^b^	4 (1.89)	3 (1.59)	1 (4.35)	
Anemia, n (%)				0.179
No	111 (52.36)	102 (53.97)	9 (39.13)	
Yes	101 (47.64)	87 (46.03)	14 (60.87)	
Radiotherapy and chemotherapy, n (%)				0.041*
No	156 (73.58)	135 (71.43)	21 (91.30)	
Yes	56 (26.42)	54 (28.57)	2 (8.70)	
Previous abdominal surgery, n (%)				0.017*
No	179 (84.43)	164 (86.77)	15 (65.22)	
Yes	33 (15.57)	25 (13.23)	8 (34.78)	
Epidural anesthesia, n (%)				0.374
No	186 (87.74)	164 (86.77)	22 (95.65)	
Yes	26 (12.26)	25 (13.23)	1 (4.35)	
Laparoscopy-assisted operation, n (%)				0.001**
No	116 (54.72)	96 (50.79)	20 (86.96)	
Yes	96 (45.28)	93 (49.21)	3 (13.04)	
Enterostomy, n (%)				0.961
No	179 (84.43)	159 (84.13)	20 (86.96)	
Yes	33 (15.57)	30 (15.87)	3 (13.04)	
Combined resection, n (%)				0.664
No	193 (91.04)	171 (90.48)	22 (95.65)	
Yes	19 (8.96)	18 (9.52)	1 (4.35)	
WBC > 10 G/L, n (%)				1.000
No	190 (89.62)	169 (89.42)	21 (91.30)	
Yes	22 (10.38)	20 (10.58)	2 (8.70)	
Hypoalbuminemia, n (%)				0.088
No	171 (80.66)	156 (82.54)	15 (65.22)	
Yes	41 (19.34)	33 (17.46)	8 (34.78)	
CEA > 9.7 ng/mL, n (%)				0.038*
No	165 (77.83)	151 (79.89)	14 (60.87)	
Yes	47 (22.17)	38 (20.11)	9 (39.13)	
CA199 > 37 U/mL, n (%)				0.276
No	178 (83.96)	161 (85.19)	17 (73.91)	
Yes	34 (16.04)	28 (14.81)	6 (26.09)	
ASA score, n (%)				0.809
≦2	161 (75.94)	144 (76.19)	17 (73.91)	
>2	51 (24.06)	45 (23.81)	6 (26.09)	
NSR2002score, n (%)				0.266
<3	193 (91.04)	174 (92.06)	19 (82.61)	
≧3	19 (8.96)	15 (7.94)	4 (17.39)	
CCI score, n (%)				0.251
0	116 (54.72)	106 (56.08)	10 (43.48)	
≧1	96 (45.28)	83 (43.92)	13 (56.52)	
Estimated blood loss > 300 ml, n (%)				1.000
No	196 (92.45)	175 (92.59)	21 (91.30)	
Yes	16 (7.55)	14 (7.41)	2 (8.70)	
Clavien-Dindo classification, n (%)				0.021*
0-II	146 (68.87)	135 (71.43)	11 (47.83)	
III-V	66 (31.13)	54 (28.57)	12 (52.17)	

*BMI* body mass index, *TNM* Tumor Node Metastasis, *ASA* American Society of Anesthesiology, *NRS2002* Nutritional Risk Screening 2002, *CCI* Charlson Comorbidity Index, *WBC* White Blood Cell, *CEA* Carcino Embryonic Antigen, *CA199* Cancer Antigen 199.

*p < 0.05, ** *p* < 0.01

^a^Undifferentiated carcinomas include poorly differentiated adenocarcinomas, signet ring cell carcinomas, and mucinous carcinomas.

^b^Differentiated carcinomas include well or moderately differentiated, tubular or papillary adenocarcinomas.

### Association of DROSD and CRC patient prognosis

**Fig. 2:**
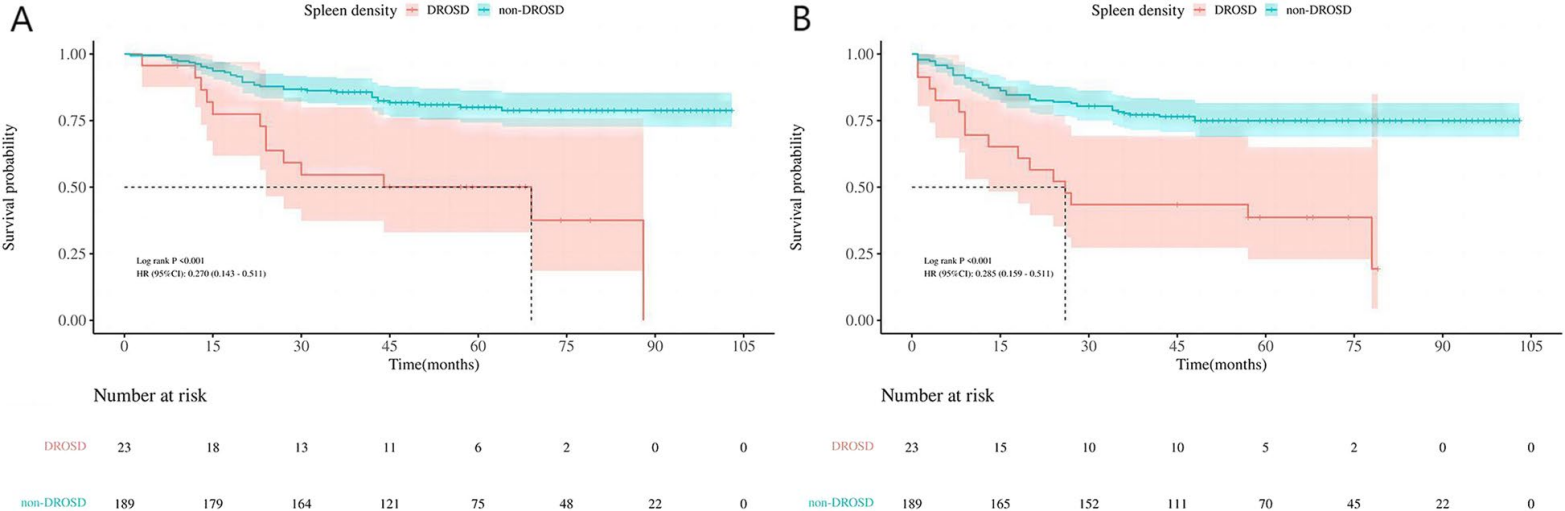
**A** Kaplan–Meier survival curves for overall survival in patients with and in those without DROSD. **B** Kaplan–Meier survival curves for disease-free survival in patients with and in those without DROSD.Two curves were compared using log-rank test. DROSD: Diffuse reduction of spleen density

**Fig. 3:**
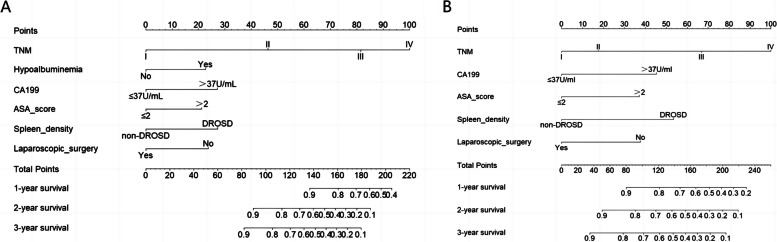
Nomogram model predicting 1-, 2- and 3-year OS and DFS in colorectal cancer patients. To utilize the nomogram, begin by assigning points to each variable of the patient through drawing a vertical line extending up to the top line labeled "Points". Calculate the cumulative points for all risk factors as total points. Subsequently, draw a vertical line downwards from the axis marked "Total Points" until reaching the bottom line in order to obtain the predicted probability of 1-, 2-, and 3-year survival following colorectal cancer resection. **A** The nomogram predicted 1-, 2- and 3-year overall survival (OS) for colorectal cancer patients. **B** The nomogram predicted 1-, 2- and 3-year disease-free survival (DFS) for colorectal cancer patients

As shown in Fig. 2, patients with DROSD had a poorer OS rate than those without DROSD (*P* < 0.001). The 1-, 2- and 3-year OS rates were 91.10%, 63.80% and 54.70%, respectively, for patients with DROSD, and were 96.30%, 87.80% and 85.70%, respectively, for those without DROSD. The median OS was shorter in patients with DROSD than in those without DROSD [HR 0.27 95%CI (0.14–0.51) Log rank *P* < 0.001]. Moreover, patients with DROSD had a poorer DFS rate than those without DROSD (*P* < 0.001). The 1-, 2- and 3-year DFS rates were 69.60%, 52.20% and 43.50%, respectively, for patients with DROSD, and were 88.40%, 82.00% and 77.70%, respectively, for those without DROSD. The median DFS was shorter in patients with DROSD than in those without DROSD [HR 0.29 95%CI (0.16–0.51) Log rank *P* < 0.001; Fig. 3].

### Univariable and multivariable analyses identify prognostic factors

Univariable analysis demonstrated that TNM stage, laparoscopy-assisted operation, hypoalbuminemia, CEA, CA199, ASA score, CCI score, Clavien-Dindo classification and DROSD were prognostic predictors for OS (*P* < 0.05) (Table 2). Multivariable analysis indicated that DROSD [HR 2.36 95%CI (1.21–4.63) *P* = 0.012], CA199 > 37 U/mL [HR 2.36 95%CI (1.25–4.46) *P* = 0.008], laparoscopy-assisted operation [HR 0.47 95%CI (0.23–0.97) *P* = 0.04], hypoalbuminemia [HR 2.05 95%CI (1.05–4.00) *P* = 0.036], ASA score > 2 [HR 1.95 95%CI (1.09–3.50) *P* = 0.025] and TNM stage III-IV [stage III HR 13.16 95%CI (1.75–98.99) *P* = 0.012; stage IV HR 23.69 95%CI (2.71–206.94) *P* = 0.004] were independent prognostic factors for OS (Table 2).

**Table 2:** Univariate and multivariate analyses of the factors affecting overall survival (OS) by Cox proportional hazard model

Factors	Univariate analysis		Multivariate analysis	
	***P*-values**	**HR (95%CI)**	***P*-values**	**HR (95%CI)**
Age	0.155	1.02 (0.99—1.05)		
BMI	0.304	0.95 (0.87—1.04)		
Surgical duration	0.367	1.00 (1.00—1.01)		
Length of postoperative hospital stay	0.143	1.01 (1.00—1.03)		
Tumor size				
<4		Ref		
≧4	0.159	1.58 (0.84—2.98)		
Gender				
Male		Ref		
Female	0.691	0.89 (0.49—1.61)		
Tumor location				
Rectum		Ref		
Colon	0.743	1.10 (0.63—1.93)		
TNM				
I		Ref		Ref
II	0.104	5.35 (0.71—40.39)	0.156	4.33 (0.57—32.79)
III	0.010*	13.81 (1.87—101.79)	0.012*	13.16 (1.75—98.99)
IV	0.002**	26.80 (3.21—223.45)	0.004**	23.69 (2.71—206.94)
Histologic type				
Differentiated^a^		Ref		
Undifferentiated^b^	0.894	0.87 (0.12—6.34)		
Anemia				
No		Ref		
Yes	0.262	1.38 (0.79—2.42)		
Radiotherapy and chemotherapy				
No		Ref		
Yes	0.980	1.01 (0.53—1.90)		
Previous abdominal surgery				
No		Ref		
Yes	0.327	1.42 (0.71—2.84)		
Epidural anesthesia				
No		Ref		
Yes	0.125	0.33 (0.08—1.36)		
Laparoscopy-assisted operation				
No		Ref		Ref
Yes	0.006**	0.40 (0.21—0.76)	0.040*	0.47 (0.23—0.97)
Enterostomy				
No		Ref		
Yes	0.518	1.27 (0.62—2.62)		
Combined resection				
No		Ref		
Yes	0.804	1.12 (0.45—2.84)		
WBC > 10 G/L				
No		Ref		
Yes	0.118	1.83 (0.86—3.91)		
Hypoalbuminemia				
No		Ref		Ref
Yes	0.034*	1.96 (1.05—3.64)	0.036*	2.05 (1.05—4.00)
CEA > 9.7 ng/mL				
No		Ref		
Yes	0.012*	2.13 (1.18—3.84)		
CA199 > 37 U/mL				
No		Ref		Ref
Yes	< 0.001**	3.25 (1.78—5.91)	0.008**	2.36 (1.25—4.46)
ASA score				
≦2		Ref		Ref
>2	0.002**	2.48 (1.39—4.41)	0.025*	1.95 (1.09—3.50)
NSR2002 score				
<3		Ref		
≧3	0.078	2.06 (0.92—4.59)		
CCI score				
0		Ref		
≧1	0.013*	2.07 (1.16—3.66)		
Estimated blood loss > 300 ml				
No		Ref		
Yes	0.598	0.73 (0.23—2.35)		
Clavien-Dindo classification				
0-II		Ref		
III-V	0.001**	2.56 (1.46—4.49)		
Spleen density				
non-DROSD		Ref		Ref
DROSD	< 0.001**	3.70 (1.96—6.98)	0.012*	2.36 (1.21—4.63)

*BMI* Body mass index, *TNM* Tumor Node Metastasis, *ASA* American Society of Anesthesiology, *NRS2002* Nutritional Risk Screening 2002, *CCI* Charlson Comorbidity Index, *WBC* White Blood Cell, *CEA* Carcino Embryonic Antigen, *CA199* Cancer Antigen 199.

*p < 0.05, ** *p* < 0.01

^a^Undifferentiated carcinomas include poorly differentiated adenocarcinomas, signet ring cell carcinomas, and mucinous carcinomas.

^b^Differentiated carcinomas include well or moderately differentiated, tubular or papillary adenocarcinomas.

The univariable analysis for DFS revealed that TNM stage, laparoscopy-assisted operation, CEA, CA199, ASA score, CCI score, DROSD and Clavien-Dindo classification were prognostic factors for DFS (*P* < 0.05) (Table 3). Multivariable analysis indicated that DROSD [HR 2.58 95%CI (1.39–4.79) *P* = 0.003], CA199 > 37 U/mL [HR 2.17 95%CI (1.20–3.94) *P* = 0.01], laparoscopy-assisted operation [HR 0.53 95%CI (0.29–0.96) *P* = 0.037], ASA score > 2 [HR 2.02 95%CI (1.19–3.45) *P* = 0.01] and TNM stage III-IV [stage III HR 3.39 95%CI (1.15–10.00) *P* = 0.027; stage IV HR 4.20 95%CI (1.03–17.16) *P* = 0.046] were independent prognostic factors for DFS (Table 3).

**Table 3:** Univariate and multivariate analyses of the factors affecting disease-free survival (DFS) by Cox proportional hazard model

Factors	Univariate analysis		Multivariate analysis	
	***P*-values**	**HR (95%CI)**	***P*-values**	**HR (95%CI)**
Age	0.185	1.02 (0.99—1.04)		
BMI	0.605	0.98 (0.91—1.06)		
Surgical duration	0.346	1.00 (1.00—1.01)		
Length of postoperative hospital stay	0.101	1.01 (1.00—1.03)		
Tumor size				
<4		Ref		
≧4	0.641	1.14 (0.67—1.94)		
Gender				
Male		Ref		
Female	0.520	0.84 (0.49—1.44)		
Tumor location				
Rectum		Ref		
Colon	0.611	0.88 (0.53—1.45)		
TNM				
I		Ref		Ref
II	0.291	1.78 (0.61—5.18)	0.678	1.26 (0.42—3.75)
III	0.009**	4.02 (1.41—11.44)	0.027*	3.39 (1.15—10.00)
IV	0.001**	7.57 (2.21—25.89)	0.046*	4.20 (1.03—17.16)
Histologic type				
Differentiated^a^		Ref		
Undifferentiated^b^	0.859	0.84 (0.12—6.03)		
Anemia				
No		Ref		
Yes	0.561	1.16 (0.70—1.92)		
Radiotherapy and chemotherapy				
No		Ref		
Yes	0.945	0.98 (0.55—1.73)		
Previous abdominal surgery				
No		Ref		
Yes	0.066	1.75 (0.96—3.18)		
Epidural anesthesia				
No		Ref		
Yes	0.171	0.49 (0.18—1.36)		
Laparoscopy-assisted operation				
No		Ref		Ref
Yes	0.006**	0.46 (0.27—0.81)	0.037*	0.53 (0.29—0.96)
Enterostomy				
No		Ref		
Yes	0.430	1.30 (0.68—2.50)		
Combined resection				
No		Ref		
Yes	0.529	1.29 (0.59—2.83)		
WBC > 10 G/L				
No		Ref		
Yes	0.052	1.96 (0.99—3.86)		
Hypoalbuminemia				
No		Ref		Ref
Yes	0.068	1.70 (0.96—3.01)	0.121	1.62 (0.88—2.97)
CEA > 9.7 ng/mL				
No		Ref		Ref
Yes	0.004**	2.15 (1.27—3.65)	0.103	1.66 (0.90—3.05)
CA199 > 37 U/mL				
No		Ref		Ref
Yes	< 0.001**	2.88 (1.66—5.01)	0.010*	2.17 (1.20—3.94)
ASA score				
≦2		Ref		Ref
>2	0.002**	2.29 (1.36—3.85)	0.010*	2.02 (1.19—3.45)
NSR2002 score				
<3		Ref		
≧3	0.134	1.77 (0.84—3.72)		
CCI score				
0		Ref		
≧1	0.009**	1.97 (1.18—3.28)		
Estimated blood loss > 300 ml				
No		Ref		
Yes	0.972	1.02 (0.41—2.54)		
Clavien-Dindo classification				
0-II		Ref		
III-V	0.001**	2.29 (1.38—3.79)		
Spleen density				
non-DROSD		Ref		Ref
DROSD	< 0.001**	3.51 (1.96—6.31)	0.003**	2.58 (1.39—4.79)

*BMI* Body mass index, *TNM* Tumor Node Metastasis, *ASA*, American Society of Anesthesiology, *NRS2002* Nutritional Risk Screening 2002, *CCI* Charlson Comorbidity Index, *WBC* White Blood Cell, *CEA* Carcino Embryonic Antigen, *CA199* Cancer Antigen 199.

*p < 0.05, ** *p* < 0.01

^a^Undifferentiated carcinomas include poorly differentiated adenocarcinomas, signet ring cell carcinomas, and mucinous carcinomas.

^b^Differentiated carcinomas include well or moderately differentiated, tubular or papillary adenocarcinomas.

### Creation of a nomogram to predict OS and DFS

**Fig. 4:**
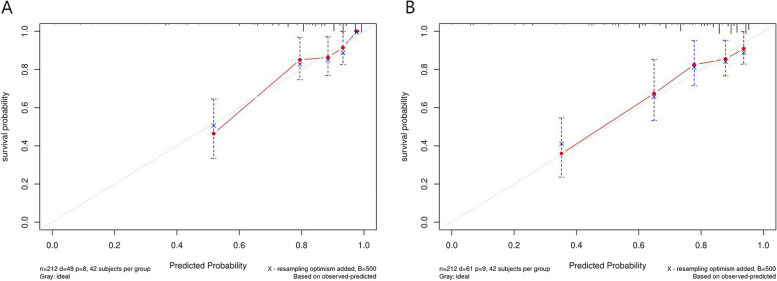
**A** Calibration curve for predicting the 3-year overall survival (OS) rates. **B** Calibration curve for predicting the 3-year overall survival disease-free survival (DFS) rates

**Fig. 5:**
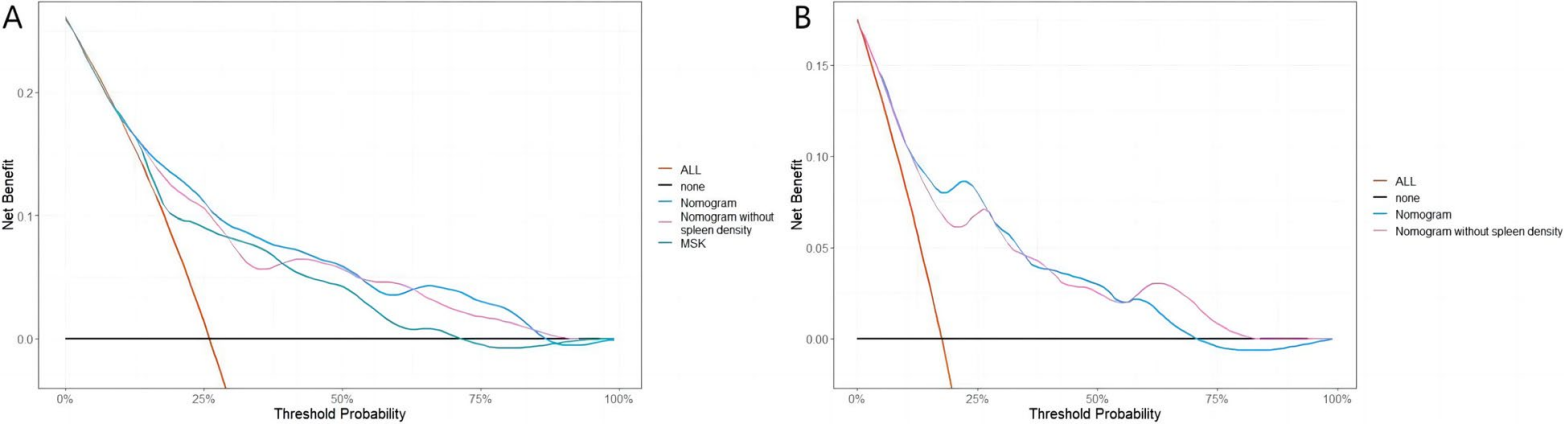
Decision curve analysis (DCA) for the nomogram.Threshold probability and net benefit were represented by the X-axis and the Y-axis, respectively. Orange line (ALL): “all patient dead scheme.” Black line (none): “no patient dead scheme.”The area between the “orange line” and “black line” in the DCA curve indicated the clinical utility of the model. **A** 3-year DFS by DCA. Green line: MSK model. Blue line: nomogram model. Purple line: nomogram without spleen density model. **B** 3-year OS by DCA. Blue line: nomogram model. Purple line: nomogram without spleen density model

A prognostic nomogram was constructed based on the independent factors of OS and DFS identified by multivariate Cox regression analysis to predict 1 -, 2 -, and 3-year survival. The nomogram demonstrated that DROSD and TNM stage were the main weighting factors in the scoring system (Fig. 3). The calibration curves showed good consistency between the predictions and observations in the 3-year OS and DFS probabilities (Fig. 4). The decision curve analysis (DCA) indicated that more net benefits within the most of thresholds probabilities were achieved using the nomogram (Fig. 5). Furthermore, we have employed the well-established risk calculator (Memorial Sloan Kettering model) to contrast it with our nomogram and have depicted the DCA in Fig. 5A. Our analysis indicates that our nomogram model provides a greater clinical net benefit in predicting 3-year DFS compared to the Memorial Sloan Kettering (MSK) model. Unfortunately, the MSK model does not offer a tool specifically for predicting 3-year OS, which precludes a direct comparison for this particular metric. Moreover, we constructed a nomogram that excludes spleen density to evaluate its contribution to the model's predictive power. Our findings demonstrate that spleen density has a positive impact on predicting 3-year DFS, as evident in Fig. 5A. Additionally, when assessing 3-year OS predictions, the addition of spleen density displays a modest improvement in the model's performance, as shown in Fig. 5B. In addition, the bootstrap-corrected C-index of the DFS and OS nomogram were found to be 0.74 (95%CI, 0.67–0.80) and 0.77 (95%CI, 0.71–0.84) respectively. Time-dependent C-index analysis also showed that the nomogram model exhibited good prognostic accuracy in clinical outcome prediction (Supplementary Fig. 1).

### Subgroup analysis

We conducted subgroup analyses to further evaluate the role of spleen density in predicting 3-year DFS and 3-year OS (supplementary Fig. 2). Variables with *P*-value < 0.1 in univariate Cox regression analysis were included in subsequent subgroup analyses. Subgroup forest plots revealed that DROSD exhibited worse 3-year DFS across various patient characteristics, including CEA level, CA199 < 37U/ml, ASA score, NSR2002 score < 3, CCI score ≧1, CD classification, hypoalbuminemia, history of previous abdominal surgery, absence of laparoscopic surgery, and TNM stage II/III. Similarly, DROSD showed worse 3-year OS in patients with characteristics such as CEA level, CA199 level, ASA score ≦2, NSR2002 score < 3, CCI score ≧1, CD classification, absence of hypoalbuminemia, history of previous abdominal surgery, absence of laparoscopic surgery, and TNM stage II/III.

## Discussion

To the best of our knowledge, this is the first study to investigate the impact of DROSD on the prognosis of patients undergoing curative colorectal cancer resection. Current research has identified several indicators exist to forecast the prognosis of colorectal cancer, with the most traditional approach being TNM stage.

TNM stage, as a well-established evaluation index, also plays a crucial role in determining the likelihood of tumor recurrence and prognosis. Similarly, our study showed that TNM stage III-IV were independent prognostic factors for OS and DFS in colorectal cancer. However, the acquisition of TNM stage necessitates post-tumor resection pathological examination, which is hindered by the drawback of delayed assessment. Consequently, enhancing the prognostic prediction and implementing hierarchical management at an early stage for patients with colorectal cancer will ameliorate the survival rate. Conversely, employing CT measurement of spleen density as an imaging modality offers expeditious, noninvasive, and cost-effective advantages. Furthermore, abdominal CT scans serve as a customary preoperative assessment for colorectal cancer without incurring supplementary expenses or subjecting patients to radiation exposure. Spleen density, as a new tool, has been shown to be a new indicator of patient prognosis [11–14]. Our study demonstrates that DROSD serves as an independent predictor for OS and DFS in this patient population. The prevalence of DROSD in our cohort of colorectal cancer patients was 10.85%. However, Deng et al. [13] observed a higher prevalence in a cohort of intrahepatic cholangiocarcinoma and Huang et al. [12] observed a higher prevalence in a cohort of gastric cancer, which were 32.9% and 24.8%, respectively. We hypothesize that the disparity in prevalence rates may be attributed to the advanced age of our cohort compared to the average age of the other cohorts [69.45 ± 10.63 VS 63.35 ± 8.55, 65 (58–73)]. Possible causes are as follows: The ratio of white pulp to red pulp in the spleen elevates with advancing age. Macrophages in the red pulp aid in the retention of red blood cells, consequently augmenting the blood volume [17]. The spleen density measured on CT is determined by its own physical density [18]. Okuma et al. [19] discovered that a reduction in spleen blood volume leads to the spleen becoming more compact, thereby increasing spleen density. In other words, as a person ages, the blood volume of the spleen decreases and the spleen becomes more compact, resulting in higher spleen density. Consequently, the percentage of DROSD declines with age.

Our study presents novel findings indicating a detrimental impact of DROSD on the prognosis of colorectal cancer patients who have undergone radical resection. The underlying mechanism linking DROSD to poor prognosis remains uncertain. Possible explanations for this association can be elucidated as follows. Firstly, the spleen, being the largest lymphoid organ in the human body, harbors a significant population of T lymphocytes, B lymphocytes, and other immune cells, thereby exerting a crucial role in maintaining immune homeostasis. However, quantifying the precise function of the spleen poses challenges [20]. Deschoolmeester et al. [21] have demonstrated that CD8^+^ T lymphocytes serve as a positive prognostic factor for OS and DFS in patients diagnosed with colorectal cancer. In certain studies investigating experimental severe acute pancreatitis in rats, it was observed that DROSD was accompanied by a significant reduction in the quantity of CD8^+^ lymphocytes within both the spleen and peripheral blood [11, 22]. Consequently, we propose the hypothesis that DROSD induces the down-regulation of splenic immune function, resulting in a decrease in the number of CD8^+^ T lymphocytes, ultimately leading to poorer OS and DFS outcomes in colorectal cancer patients. Secondly, recent studies have demonstrated that an increase in spleen volume is an independent factor associated with poor prognosis in malignant tumors [23, 24]. It was recently reported that patients with splenic volume increase have greater numbers of spleen CD4 regulatory T cells and programmed death ligand 1-and ligand 2-expressing cells than those without splenic volume increase, suggesting that patients with splenic volume increase have poorer tumor immunity than those without it [25]. Furthermore, one study has indicated a correlation between the reduction in spleen density and the increase in spleen volume in rats with severe acute pancreatitis [11]. In a similar vein, our hypothesis posited that colorectal cancer patients with DROSD would exhibit an increased spleen volume. Additionally, it has been theorized that DROSD is a result of fatty infiltration in the spleen, akin to fatty liver, which is linked to lipid metabolism [26]. Previous research has indicated that enhanced lipid metabolism and obesity are associated with a poorer prognosis in colorectal cancer [27]. Nevertheless, in the present study, BMI demonstrated minimal correlation with OS and DFS (*P* > 0.05). Even the previous literature has pointed out that BMI and obesity are protective factors for the long-term prognosis of colorectal cancer patients [28, 29].

Our study also discovered that laparoscopy-assisted surgery, elevated CA199 levels, and high ASA scores were autonomous risk factors for poorer OS and DFS in patients with colorectal cancer. CA199, as a conventional serum diagnostic marker, effectively reflects tumor activity and invasion, and has been established as an independent prognostic factor for gastric cancer [30], pancreatic ductal adenocarcinoma [31], and colorectal cancer [32]. Furthermore, our observation that hypoproteinemia independently predicts worse OS following colorectal cancer surgery aligns with the findings of Gonzalez-Trejo et al. [33]. The utilization of laparoscopic-assisted surgery for colorectal cancer has witnessed a notable surge in popularity in recent years, primarily due to the widespread adoption of laparoscopic surgery [34]. A recent study has demonstrated that laparoscopic surgery offers a reduction in postoperative complications and an improvement in survival rates when compared to open surgery, particularly among elderly patients with colorectal cancer [35]. Furthermore, certain scholarly sources indicate that a high ASA score, which serves as a risk indicator for severe postoperative complications, also diminishes survival rates [36, 37].

Physicians frequently employ statistical prediction tools such as nomograms to forecast mortality rates [38]. In this study, a DFS nomogram was developed through multivariate COX regression analysis, incorporating variables such as DROSD, CA199, TNM stage, laparoscopy-assisted operation, and ASA score. Similarly, an OS nomogram was constructed, including DROSD, CA199, TNM stage, hypoproteinemia, laparoscopy-assisted operation, and ASA score, to predict survival rates at 1, 2, and 3 years. Notably, the nomogram survival prognosis prediction method exhibits superior accuracy compared to the conventional TNM tumor stage system [39, 40]. In addition, we use calibration curves, DCA, and bootstrap resamplling method to confirm that the nomogram we build has good performance.

The current investigation was subject to several limitations. Firstly, we analysed a limited number of patients from a single institution. Secondly, our follow-up period was relatively short. Thirdly there might have been some biases that influenced spleen density and patients’ prognosis because this study was a retrospective analysis. Lastly, we have several hypotheses to elucidate this phenomenon, yet further research is paramount to substantiate it.

## Conclusions

DROSD emerges as an autonomous risk factor for OS and DFS in patients with colorectal cancer who undergo radical resection. The nomogram demonstrated good performance, particularly in predicting 3-year DFS with a net clinical benefit superior to well-established risk calculator.

### Supplementary Information

**Supplementary Material 1. **

## Data Availability

The datasets used during the current study are available from the corresponding author on reasonable request.

## Abbreviations

CRC: Colorectal cancer
DROSD: Diffuse reduction of spleen density
OS: Overall survival
DFS: Disease-free survival
CT: Computed tomography
HU: Hounsfield units
BMI: Body mass index
TNM: Tumor Node Metastasis
ASA: American Society of Anesthesiology
NRS2002: Nutritional Risk Screening 2002
CCI: Charlson Comorbidity Index
WBC: White Blood Cell
CEA: Carcino Embryonic Antigen
CA199: Cancer Antigen 199
IQR: Interquartile range
