# Supplementary material for: Correlation between spleen density and prognostic outcomes in patients with colorectal cancer after curative resection
Source: BMC Cancer. 2024 Apr 6;24:425. doi: 10.1186/s12885-024-12208-7 (PMC10999091; doi:10.1186/s12885-024-12208-7)
Supplement: Supplementary file 1 — Supplementary Material 1. [file 12885_2024_12208_MOESM1_ESM.zip › Supplementary material.docx]

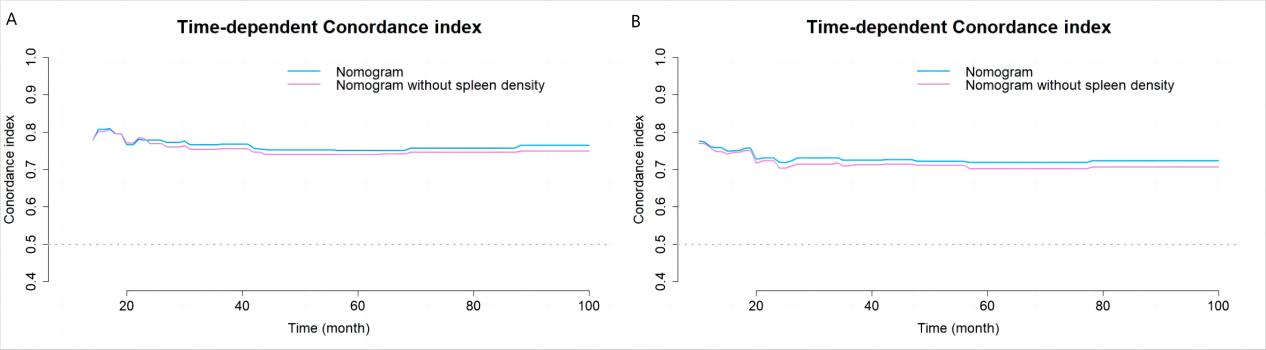


Supplementary Fig. 1 Time-dependent C-index of nomogram model compared with nomogram without spleen density model for OS (A) and DFS (B) of colorectal cancer patients using the bootstrap resampling method. Blue line : nomogram model. Purple line: nomogram without spleen density model.


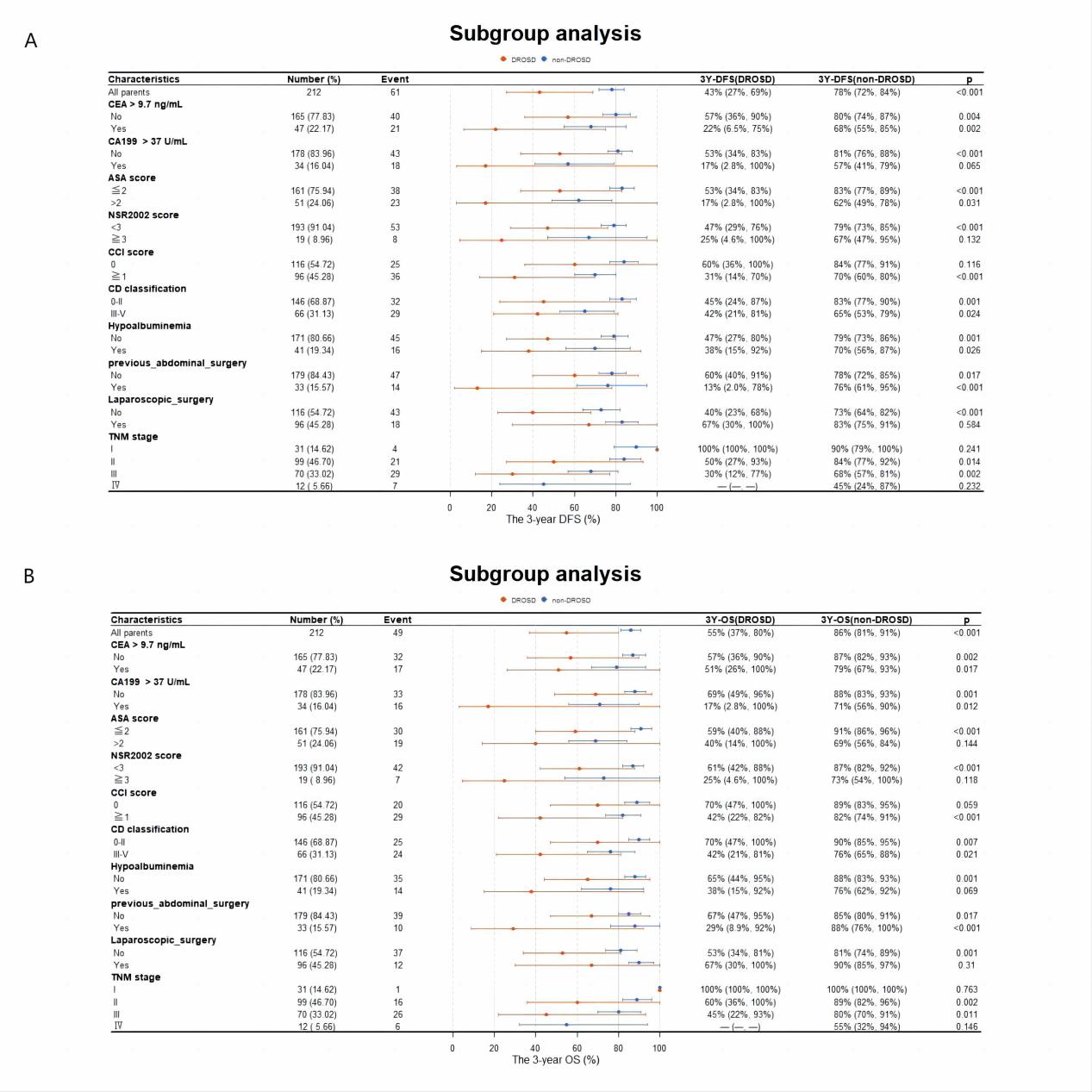


Supplementary Fig. 2 Forest plot of subgroup analysis of spleen density in colorectal cancer patients with regard to 3-year DFS (A) and 3-year OS (B).
