# Supplementary figures and images for: Correlation between spleen density and prognostic outcomes in patients with colorectal cancer after curative resection
Source: BMC Cancer. 2024 Apr 6;24:425. doi: 10.1186/s12885-024-12208-7 (PMC10999091; doi:10.1186/s12885-024-12208-7)

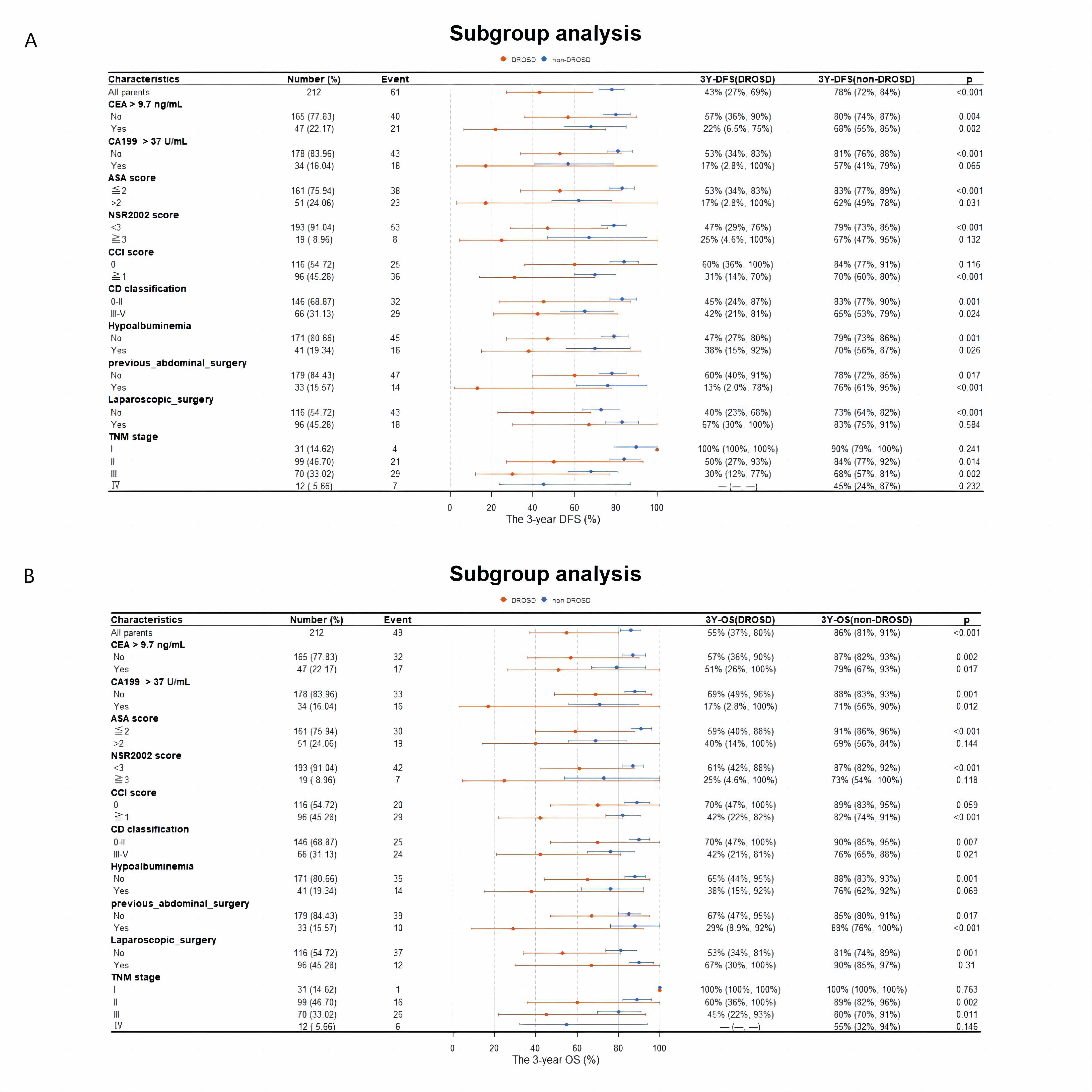

Supplement: Supplementary file 1 — Supplementary Material 1. [file 12885_2024_12208_MOESM1_ESM.zip › Supplementary Fig. 2.jpg]

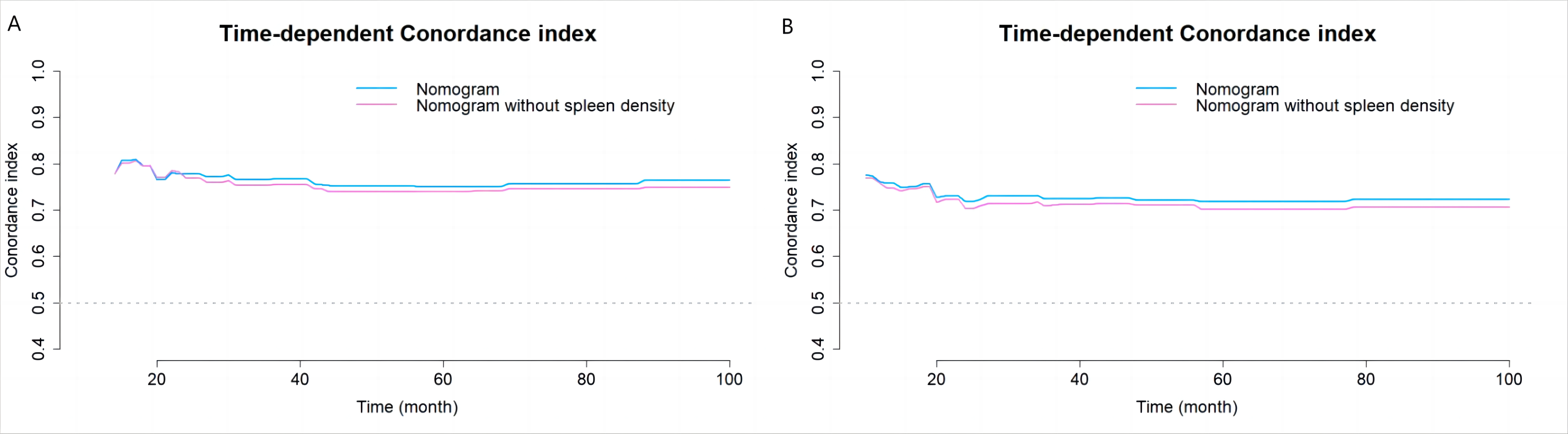

Supplement: Supplementary file 1 — Supplementary Material 1. [file 12885_2024_12208_MOESM1_ESM.zip › Supplementary Fig.1.jpg]
